# Coronavirus disease 2019 (COVID-19): Secondary bacterial infections and the impact on antimicrobial resistance during the COVID-19 pandemic

**DOI:** 10.1017/ash.2022.253

**Published:** 2022-07-11

**Authors:** Yelena Markovskaya, Elizabeth M. Gavioli, Jaclyn A. Cusumano, Aaron E. Glatt

**Affiliations:** 1 Department of Medicine, Mount Sinai South Nassau, Oceanside, NY, United States; 2 Icahn School of Medicine at Mount Sinai, New York, New York; 3 Department of Pharmacy Practice, Arnold and Marie Schwartz College of Pharmacy and Health Sciences, Brooklyn, New York; 4 Department of Pharmacy, Mount Sinai Queens, Queens, New York

## Abstract

Secondary bacterial infections and bacterial coinfections are an important complication of coronavirus disease 2019 (COVID-19), leading to antibiotic overuse and increased rates of antimicrobial resistance (AMR) during the COVID-19 pandemic. In this literature review, we summarize the reported rates of secondary bacterial infections and bacterial coinfections in patients with COVID-19, the impact on patient outcomes, the antibiotic treatment approaches employed, and the resistance patterns observed. The reported data suggest that although the incidence of secondary bacterial infections or bacterial coinfections is relatively low, they are associated with worse outcomes such as prolonged hospitalization, intensive care unit admission, mechanical ventilator use, and increased mortality. Interestingly, antibiotic prescription rates are typically higher than secondary bacterial and bacterial coinfection rates, and reports of AMR are common. These findings highlight the need for an improved understanding of secondary bacterial and bacterial coinfection in patients with COVID-19, as well as improved treatment options, to mitigate inappropriate antibiotic prescribing and AMR.

Viral pandemics have historically been associated with secondary bacterial infections, and coronavirus disease 2019 (COVID-19) has been no exception. Subsequent bacterial infections, particularly lower respiratory tract infections, which are the leading cause of infectious disease mortality worldwide,^1,2^ have been associated with increased mortality both during the 1918 Spanish influenza pandemic and during seasonal influenza outbreaks.^3,4^ However, differentiating viral versus bacterial infection is a challenge for clinicians, which has led to inappropriate or prolonged use of antibiotics in patients with COVID-19. As previously described, the overuse of antibiotics increases the risk of antimicrobial resistance (AMR)^5–7^ which can cause severe infections and complications, such as disruption of the gut microbiota leading to outbreaks of *Clostridium difficile* infection.^8,9^

In this review, we examined the prevalence of secondary bacterial infections and bacterial coinfections in patients with COVID-19 and the use of antibiotics associated with these infections. A literature search of PubMed and Embase was conducted to identify relevant studies published up to June 2, 2021 (Supplementary Table 1). The main types of bacterial infections studied were (1) coinfections or community-acquired (CA) infections prior to or within the first 3 d of hospitalization, (2) secondary or hospital-acquired (HA) infections on or after day 4 of hospitalization, according to the National Healthcare Safety Network definition,^10^ and (3) both CA and HA infections.

We also reviewed the impact of secondary bacterial infections and bacterial coinfections on clinical outcomes (eg, length of hospitalization, intensive care unit [ICU] admission and mortality), the etiology of these bacterial infections, the antibiotic treatment approaches, and discuss the development of AMR.

## Prevalence of secondary bacterial infections and bacterial coinfections in patients with COVID-19

Most studies have reported an estimated rate of secondary bacterial infections and bacterial coinfections <20% (Tables 1–3).^11–16^ However, CA bacterial infection has been less commonly reported, with rates ranging between 1% and 7.5% (Table 1).^13,14,17,18^ The rates of HA bacterial infections were variable and ranged from 9.3% to 32% for overall secondary bacterial infections (Table 3).^19,20^ Although the heterogeneity of the methodologies and populations (eg, moderate-to-severe COVID-19 cases, outpatients vs inpatients) make it difficult to compare rates of bacterial infections, in general, HA infection rates tended to be higher than CA infection rates in studies that recorded data on both (Table 2).^15,16,21,22^

**Table 1.** Summary of Included Studies Involving Patients With COVID-19 and Community-Acquired (CA) Bacterial Coinfections

Study	Study Type/Date	Total COVID-19 Patients, No.	Coinfection Type^a^/Acquisition Setting	Time of Infection Diagnosis^a^	Rate of Coinfection^b^	Mortality Outcomes in COVID-19 Patients With Coinfection^c^	Other Outcomes Reported in COVID-19 Patients With or Confection
**Observational studies, United States**							
Vaughn et al, 2020^14^	Retrospective cohort, 8 hospitals, USA (March–June 2020)	1,705	CA bacterial infection/outpatient	≤3 d of hospitalization	59 (3.5%) of 1,705 CA bacterial infections; bacterial respiratory infection, 29 (1.7%) of 1,705; BSI, 31 (1.8%) of 1,705	Mortality rate, 28 (47.5%) of 71 in patients with CA bacterial infections vs 297 (18.0%) of 1,634 w/o confirmed infection (*P* < .001)	Hospital LOS
Karaba et al, 2020^13^	Retrospective cohort, 5 hospitals, USA (March–May 2020)	1,016	CABP/outpatient	≤48 h of admission	Proven/probable CABP, 12 (1.2%) of 1,016; nonrespiratory coinfection, 42 (4%) of 1,016	NR	Hospital LOS, ICU admission rate
Observational studies: Europe							
Thelen et al, 2021^17^	Retrospective cohort, 2 hospitals, Netherlands (February–June 2020)	678	CA bacterial infection/outpatient	≤48 h of COVID-19 test	Bacteremia, 7 (1.0%) of 678	30-d all-cause mortality rate in patients with BSI, 26.2%	NR
**Observational studies: Asia**							
Wang et al, 2020^18^	Retrospective cohort, 1 hospital, China (January 2020)	67 (69 enrolled but 2 lost)	CABP/outpatient	NR	Bacterial respiratory infections: 5 (7.5%) of 67	Mortality rate, 5 (7.5%) of 67	NR^d^
Hoshyama et al, 2020^63^	Retrospective cohort, 1 hospital, Japan (February–July 2020)	7	CA bacterial infection/outpatient	On admission	Bacterial respiratory, 4 (57.1%) of 7	Mortality rate, 0%; all 7 patients were discharged	NR

Note. CABP, community-acquired bacterial pneumonia; ICU, intensive care unit; LOS, length of stay; NR, not reported; w/o, without.

a Based on published information, including clinical details, or on the time of infection diagnosis: outpatient/≤3 d of hospitalization = community acquired infection, unless otherwise stated in the source.

b Rates were reported per total number of patients with COVID-19.

c Data for hospital LOS, ICU admission rates in patients with COVID-19 who had coinfections.

d Outcomes were reported in total patient population.

**Table 2.** Summary of Included Studies Involving Patients With COVID-19 and Community-Acquired (CA) Bacterial Coinfections or Hospital-Acquired (HA) Secondary Bacterial Infections

Study	Study Type/Date	Total COVID-19 Patients, No.	Coinfection or Secondary Infection Type^a^/Acquisition Setting	Time of Infection Diagnosis^a^	Rate of Secondary Bacterial Infection or Coinfection^b^	Mortality Outcomes in COVID-19 Patients With Secondary Bacterial Infection or Coinfection^c^	Other Outcomes Reported in COVID-19 Patients With Secondary Bacterial Infection or Coinfection^c^
**Observational studies, United States**							
Singh et al, 2021^33^	Analysis of respiratory samples, 1 laboratory, USA (March–August 2020)	4,259	CA and HA respiratory bacterial infections/outpatient and inpatient	NR	Bacterial, 33.2%	NR	NR
Kubin et al, 2021^21^	Retrospective cohort, 1 hospital, USA (March–May 2020)	3,028	CA and HA bacterial/fungal infection/outpatient and inpatient	≤72 h of hospitalization or ≤5 d prior to admission from outpatient/ED visit (CA infection); after hospital day 3 (HA infection)	Overall bacterial/fungal infection, 516 (17.0%) of 3,028; CA infection, 183 (6.0%) of 3,028; HA infection, 350 (11.6%) of 3,028	Mortality rate, 168 (33%) of 516	Hospital LOS, ICU admission rate, MV rate
Cusumano et al, 2020^25^	Retrospective case series, 2 hospitals, USA (March–May 2020)	2,679; 42 with *S. aureus* bacteremia	CA and HA *Staphylococcus aureus* bacteremia/outpatient and inpatient	On admission; ≥4 d after admission (HA bacteremia)	*S. aureus* bacteremia, 42 (1.6%) of 2,679; HA-bacteremia, 28 (66.7%) of 42	14-d mortality rate, 23 (54.8%) of 42; 30-d mortality rate, 28 (66.7%) of 42	Hospital LOS
**Observational studies, Europe**							
Amin-Chowdhury et al, 2020^46^	Prospective national cohort study, England (February–June 2020)	160,886	Bacterial coinfection with IPD/outpatient and inpatient	Coinfection, ≤2 d of positive COVID test	Coinfection, 40 (0.025%) of 160,886	Mortality rate (<28 d), 25 (63.2%) of 40	NR
Russell et al, 2021^41^	Prospective cohort, 260 hospitals, UK (February–June 2020)	48,902	CA and HA-acquired bacterial infection/outpatient and inpatient	≤2 d of admission (coinfection) and ≥3 d (HA infection)	Respiratory or BSI, 1,107 (2.3%) of 48,902^g^; unrelated infections, 1,002 (2.0%) of 48,902^g^; 70.6% of infections were HA	No association between respiratory infection or BSI and mortality in ICU patients	NR
Calderón-Parra et al, 2021^53^	Retrospective cohort SEMI-COVID-19 registry, 150 hospitals, Spain (March–June 2020)	13,932	CA and HA bacterial infection/outpatient and inpatient	NR	NR	NR^d^	NR^d^
Sharov et al, 2020^26^	Two sampling sets, Russia (March–May 2020)	Set 1: 147 of 3,382 patients with COVID-19-related pneumonia; Set 2: 1,204 patients with pneumonia and COVID-19	CA and HA bacterial pneumonia/outpatient and inpatient	At admission, day 4, and day 10 of hospitalization or with clinical deterioration	Set 1: bacterial pneumonia, 61 (41.5%) of 147. Set 2: 433 (36.0%) of 1,204; HA, 239 (55.2%) of 433	Set 1: 91.7% of lethal COVID-19 cases associated with secondary bacterial pneumonia. Set 2: patients with diagnosed bacterial pneumonia, 57 (17.7%) of 322	NR
Garcia-Vidal et al, 2021^15^	Retrospective cohort, 1 hospital, Spain (February–April 2020)	989	CA bacterial infection/outpatient	≤24 h of admission	CA bacterial infections, 25 (2.5%) of 989; bacterial pneumonia, 21 (2.1%) of 989	Mortality rate, 5 (16.1%) of 31 patients with CA coinfections	Hospital LOS, ICU admission rate, ICU LOS
			HA bacterial infection/inpatient	≥48 h after admission; mean time to diagnosis, 10.6 d	HA bacterial infections, 38 (3.8%) of 989	Mortality rate, 8 (18.6%) of 43 patients with HA superinfections	Hospital LOS, ICU admission rate, ICU LOS
Hughes et al, 2021^23^	Retrospective cohort, 2 hospitals, UK (February–April 2020)	836	CA bacterial infection/outpatient	<5 d from admission (CA infection)	27 (3.2%) of 836 early bacterial infections (0–5 d after admission); bacterial CA pathogens, 14 (35.9%) of 39 among 112 respiratory samples taken	Relative risk of death of patients with true pathogens in blood against baseline of admitted patients, 1.51 (*P* = .3543)	
			HA bacterial infection/inpatient	>5 d from admission (HA infection)	51 (6.1%) of 836 bacterial infections throughout admission; bacterial HA pathogens, 25 (64.1%) of 39 among 112 respiratory samples taken		
Rouze et al, 2021^39^	Retrospective cohort, 36 ICUs, EU (March–May 2020)	568	CA and HA pneumonia infection/outpatient and inpatient	≤48 h intubation (n=359)	Overall pneumonia, 55 (9.7%) of 568; pneumonia with <48 h hospital stay, 29 (8.1%) of 359	28-d mortality rate, 24 (43.6%) of 55; increased adjusted HR for 28-d mortality, 1.57 (95% CI, 1.01–2.44; *P* =.043)	ICU LOS, ICU LOS, MV duration, ICU mortality
Baskaran et al, 2021^36^	Retrospective cohort, 7 ICUs, England, (February–May)	254	CA and HA infection/outpatient and inpatient	<48 h (CA infection), >48 h (HA infection)	Overall bacterial infection, 83 (32.7%) of 254^g^; bacterial CA, 14 (5.5%) of 254; bacterial/fungal HA, 77 (30.3%) of 254	Mortality rate, 8 of 43 (18.6%); *P* = .047 vs patients w/o HA infection	ICU LOS
Foschi et al, 2021^32^	Retrospective cohort, Italy, ICUs, (March–December 2020)	178 critically ill	CA and HA respiratory bacterial infection, mostly HA/outpatient and inpatient	NR	Respiratory bacterial infections, 79 (34.3%) of 230 samples among 178 patients	NR	NR
Søgaard et al, 2020^16^	Retrospective cohort, 1 hospital, Switzerland (February–May 2020)	162	CA and HA respiratory tract infection/outpatient and inpatient	≤48 h of admission (CA infection)	Bacterial CA pneumonia and bacteremia, 1 (0.6%) of 162; bacterial HA infection, 23 (13.6%) of 162	NR	NR
**Observational studies, Asia**							
Tan et al, 2020^59^	Antibiotic use point prevalence survey, 2 hospitals, Singapore (April 2020)	577 (554 confirmed, 23 suspected)	CA and HA infection/outpatient and inpatient	NR	NR	NR^d^	NR^d^
Chen et al, 2021^27^	Retrospective cohort, 1 hospital, China (January–March 2020)	408	CA and HA infection/outpatient and inpatient	<48 h (CA infection); >48 h (HA infection)	Bacterial/viral CA, 33 (8.1%) of 408; bacterial/fungal HA, 21 (5.1%) of 408	NR^d^	NR^d^
Nasir et al, 2021^24^	Retrospective case-control study, 1 hospital, Pakistan, (February–June 2020)	100: 50 cases with and 50 controls w/o bacterial infection	CA and HA bacterial infection/outpatient and inpatient	NR	28% CA bacterial infections and 72% HA bacterial infections. Most common infection: HA pneumonia, 28 (56%) of 50; CA pneumonia, 8 (16%) of 50	Mortality rate, 21 (42%) of 50, vs 18% w/o bacterial infection (*P* < .05)	Hospital LOS, ICU admission rate, MV rate
**Meta-analyses and reviews**							
Langford et al, 2021^52^	Systematic review/meta-analysis, 154 studies (December 2019–May 2020)	35,263	Coinfection and secondary bacterial infection/outpatient and inpatient	NR	Bacterial coinfection, 8.6% from 31 studies pooled	NR	NR
Lansbury et al, 2020^34^	Systematic review/meta-analysis, 30 studies (January–April 2020)	3,834	Coinfection and secondary infection/outpatient and inpatient	NR	Bacterial, 7% for hospitalized patients; 14% for ICU patients	Crude pooled OR for death patients with vs w/o coinfection, 5.82 (95% CI, 3.4–9.9)	NR
Vazzana et al, 2021^64^	Systematic review/meta-analysis, 355 studies (December 2019–April 2020)	3,492	CA and HA bacterial infection/outpatient and inpatient	NR	Secondary bacterial infections, 4.8%–19.5% from 8 studies pooled	Risk of severe course and/or fatal outcomes was significantly increased in patients with evidence of bacterial infection (OR, 20.8; 95% CI, 11.6–37.4)	NR
Langford et al, 2020^22^	Systematic review/meta-analysis, 24 studies (December 2019–March 2020)	3,338	Coinfection and secondary bacterial infection/outpatient and inpatient	On presentation (coinfection); emerging during illness or hospital stay (secondary infection)	Overall bacterial, 6.9%; CA, 3.5%; HA, 14.3%	NR	NR
Rawson et al, 2020^31^	Systematic review, 9 studies (January–April 2020)	806 (infection rates); 2010 (antimicrobial prescribing)	CA and HA bacterial and fungal infection/outpatient and inpatient	NR	Bacterial/fungal infection, 62 (8%) of 806	NR	NR

Note. BSI, bloodstream infections; CA, community acquired; CABP, community-acquired bacterial pneumonia; CPE, carbapenemase-producing Enterobacterales; CRKp, carbapenem-resistant *Klebsiella pneumoniae*; CRPA, carbapenem-resistant *Pseudomonas aeruginosa*; ED, emergency department; EU, European Union; HA, hospital-acquired; HAP, hospital-acquired pneumonia; ICU, intensive care unit; IPD, invasive pneumonococcal disease; LOS, length of stay; MDR, multidrug resistant; MV, mechanical ventilation; NR, not reported; OBD, occupied bed days; OR, odds ratio; patients, patients; VAP, ventilator-associated pneumonia; w/o, without.

a Based on published information, including clinical details, or on the time of infection diagnosis: outpatient/≤3 d of hospitalization = community acquired infection; ≥4 d of hospitalization = hospital-acquired infection, unless otherwise stated in the source.

b Rates were reported per total number of patients with COVID-19.

c Data for hospital LOS, ICU admission rates in patients with COVID-19 who secondary bacterial infections or coinfections.

d Outcomes were reported in total patient population.

**Table 3.** Summary of Included Studies Involving Patients With COVID-19 and Hospital-Acquired (HA) Secondary Bacterial Infections

Study	Study Type/Date	Total COVID-19 Patients, No.	Secondary Infection Type^a^/Acquisition Setting	Time of Infection Diagnosis^a^	Rate of Secondary Bacterial Infection^b^	Mortality Outcomes in COVID-19 Patients With Secondary Bacterial Infection^c^	Other Outcomes Reported in COVID-19 Patients With Secondary Bacterial Infection^d^
**Observational studies, South America**							
Martinez-Guerra et al, 2021^12^	Prospective cohort, 1 hospital, Mexico (March–June 2020)	794	HA bacterial infection/inpatient	Median hospital stay at diagnosis, 9 d	Overall infection, 74 (11.3%) of 656; VAP/HAP, 56 (50.9%) of 110 episodes; BSI, 6 (29.1%) of 110 episodes	Mortality rate, 30 (40.5%) of 74 vs 33% w/o infection (*P* < .05)	Hospital LOS
Silva et al, 2021^11^	Retrospective cohort, 1 hospital, Brazil (May–November 2020)	212	HA bacterial and fungal infection/inpatient	NR	Bacterial, 34 (16%) of 212	Increased risk of death with bacterial infection	NR
**Observational studies, United States**							
Nori et al, 2020^28^	Retrospective cohort, 1 hospital, USA (March–April 2020)	4,267	HA bacterial or fungal infection, inpatient	Time to culture positivity, 6–7 d	Bacterial/fungal, 152 (3.6%) of 4,267	Mortality rate, 68 (57%) of 152	Hospital LOS, ICU admission
Gomez-Simmonds et al, 2021^45^	Retrospective cohort, 1 hospital, USA (March–April 2020)	3,152; 13 with CPE infection	HA CPE infection/inpatient	NR	CPE, 13 (0.4%) of 3,152; respiratory, 11 (0.3%) of 3,152; bacteremia, 7 (0.2%) of 3,152	Mortality rate, 5 (38.5%) of 13	ICU admission rate and MV rate
Adelman et al, 2021^37^	Retrospective cohort, 4 hospitals, USA (February–May 2020)	774	HA bacterial pneumonia and BSIs/inpatient	NR	BSI, 36 (4.7%) of 774; respiratory, 65 (27.3%) of 238 intubated patients; VAP, 2%	Mortality rate in patients with BSI, 50%; m ortality rate in intubated patients, 41.5%	NR
Chong et al, 2021^40^	Retrospective cohort, 1 hospital, USA (March–June 2020)	244	HA pulmonary bacterial infection/inpatient	≥48 h after admission	Pulmonary, 13 (5%) of 244	No difference in mortality vs w/o infection	hospital LOS, ICU admission rate, MV rate
Obata et al, 2020^48^	Retrospective cohort, 1 hospital, USA (March–May 2020)	226: 57 received steroid; 169 w/o steroids (n=169)	HA bacterial infection/inpatient	NR	Bacterial infection in steroid group, 14 (24.6%) of 57 vs 19 (11.2%) of 169 w/o steroids	NR^d^	NR^d^
Kimmig et al, 2020^47^	Retrospective cohort, 1 ICU, USA (March–April 2020)	111: 48 treated with tocilizumab; 63 w/o tocilizumab	HA bacterial infection/inpatient	NR	Patients treated with tocilizumab: bacterial, 24 (50%) of 48, VAP/HAP, 18 (37.5%) of 48. Patients w/o tocilizumab: bacterial, 18 (28.6%) of 63, VAP/HAP, 11 (17.5%) of 63	NR^d^	NR^d^
**Observational studies, Europe**							
Ripa et al, 2020^19^	Cohort study, 1 hospital, Italy (February–April 2020)	731	HA bacterial infection/inpatient	≥48 h after admission	Overall infection, 68 (9.3%) of 731^g^; possible LRTI 22 (3.0%) of 731; BSI, 58 (7.9%) of 731	Mortality rate, 30 (44.1%) of 68 vs 24.7% w/o HA infection (*P* < .001)	ICU admission rate
Posteraro et al, 2021^35^	Retrospective cohort, 1 hospital, Italy (March–May 2020)	293; 46 with BSI	HA BSI/inpatient	>48 h after admission or after discharge from previous hospital	BSI, 46 (15.7%) of 293^g^	Mortality rate, 20 (43.5%) of 46 *vs* 52 (24.2%) of 215 w/o positive blood cultures (*P* = .008)	NR
Guisado-Gil et al, 2020^50^	Retrospective cohort, 1 hospital, Spain (March–May 2020)	282	HA candidemia and MDR BSIs/inpatient	HA, >48 h after admission	Incidence candidemia and bacterial BSI per 1,000 OBD: Q1 2020, 0.37 cases; Q2 2020, 0.24 cases	Mortality rate, 17.6% at day 14 and 26.5% at day 30 in patients with MDR BSIs	NR
Magnasco et al, 2020^51^	Retrospective cohort, 2 ICUs, Italy (February– May 2020)	118	HA drug-resistant infection^e^/inpatient	HA, 10–30 d after ICU admission	Drug resistant, 14 (11.9%) of 118; CRPA, 12 (10.2%) of 118; CR-Kp, 2 (1.6%) of 118^f^	Patients with CRPA: crude mortality rate, 5 (41.7%) of 12. Patients with CR-Kp: observed mortality rate, 1 (50%) of 2	ICU LOS
Pink et al, 2021^20^	Retrospective cohort, 1 hospital, Germany (March–October 2020)	99	HA bacterial infection/inpatient	NR	Bacterial, 32%	NR^d^	NR^d^
Bogossian et al, 2020^42^	Retrospective case control study, 1 ICU, Belgium (March–April 2020)	72	HA MDR bacterial infection/inpatient	NR	MDR bacterial, 24 (33%) of 72	ICU mortality rate, 6 (25%) of 24; hospital mortality rate, 6 (25%) of 23	Hospital LOS, ICU LOS, MV rate, MV duration
García-Meniño et al, 2021^44^	Case series, 1 ICU, Spain (February 2020)	62	HA CP-Kp infection/inpatient	4–15 d after ICU admission	CP-Kp, 7 (11.3%) of 62	Mortality rate, 1 (14.3%) of 7	MV rate
Buehler et al, 2021^30^	Prospective cohort study, 1 ICU, Switzerland (April–June 2020)	45	HA pulmonary infection/inpatient	Day 10 after ICU admission (mean)	Bacterial/fungal, 19 (42.2%) of 45	NR	ICU LOS, MV duration
Montrucchio et al, 2020^65^	Cohort study, 1 ICU, Italy (March–May 2020)	35	HA CP-Kp infection/inpatient	6–22 d after ICU admission	CP-Kp, 7 (20%) of 35	28-d mortality rate, 2 (28.6%) of 7	ICU LOS, MV duration
**Observational studies, Asia**							
Lee et al, 2021^38^	Retrospective cohort, 1 hospital Korea (February– July 2020)	140	HA infection/inpatient	5.8 ± 6.7 d after admission	Overall secondary infection, 31 (22.1%) of 140; secondary bacterial infection, 30 (21.4%) of 140	Mortality rate, 6.5% vs 0% w/o HA infection (*P* = .048)	MV rate
Yang et al, 2020^29^	Retrospective cohort, 1 ICU, China (December 2020–January 2021)	52	HA bacterial and fungal infection/inpatient	NR	Bacterial/fungal, 7 (13.5%) of 52	NR^d^	NR^d^
Fu et al, 2020^43^	Retrospective cohort, 1 ICU, China (February–April 2020)	36	HA bacterial infection/inpatient	Average time from ICU admission, 11 d	Bacterial, 5 (13.9%) of 36	Mortality rate, 1 (20%) of 5	MV rate

Note. BSI, bloodstream infections; CPE, carbapenemase-producing Enterobacterales; CR-Kp, carbapenem-resistant *Klebsiella pneumoniae*; CRPA, carbapenem-resistant *Pseudomonas aeruginosa*; ED, emergency department; EU, European Union; HA, hospital-acquired; HAP, hospital-acquired pneumonia; HFNT, high flow nasal therapy; ICU, intensive care unit; LOS, length of stay; MDR, multidrug resistant; MV, mechanical ventilation; NR, not reported; OBD, occupied bed d; OR, odds ratio; patients, patients; VAP, ventilator-associated pneumonia; w/o, without.

a Based on published information, including clinical details, or on the time of infection diagnosis: ≥4 d of hospitalization = hospital-acquired infection, unless otherwise stated in the source.

b Rates were reported per total number of patients with COVID-19.

c Data for hospital/ICU LOS, ICU admission rates, MV rates, MV duration in patients with COVID-19 who secondary bacterial infections.

d Outcomes were reported in total patient population.

Respiratory tract infections and bloodstream infections were the most common bacterial HA infections observed.^14,23^ Specifically, in a case–control study of 50 COVID-19 patients with bacterial infections, 56% had HA bacterial pneumonia versus 16% with CA pneumonia.^24^ The higher rates of HA infections may be linked to ICU admission, ventilator-associated infections, and prolonged hospital stay.^16^ Indeed, a single-center study of hospitalized patients with COVID-19 in the United States reported that ICU stay and mechanical ventilation were independent predictors of HA infection in patients hospitalized with COVID-19.^21^ In several studies the rates of HA and/or ventilator-associated pneumonia (VAP) infection were >50% (Table 3).^12,24–26^

Although we specifically looked at bacterial infections, a few studies reported rates both for bacterial and fungal infections together.^21,27–30^ Rates reported in hospitalized patients with COVID-19 varied from 3.6% up to 42.2% (Tables 2–3).^28,30^ In a meta-analysis including 9 studies, 8% of patients with COVID-19 experienced bacterial or fungal coinfections during hospital admission.^31^

## Etiology of bacterial infections

**Fig. 1.**
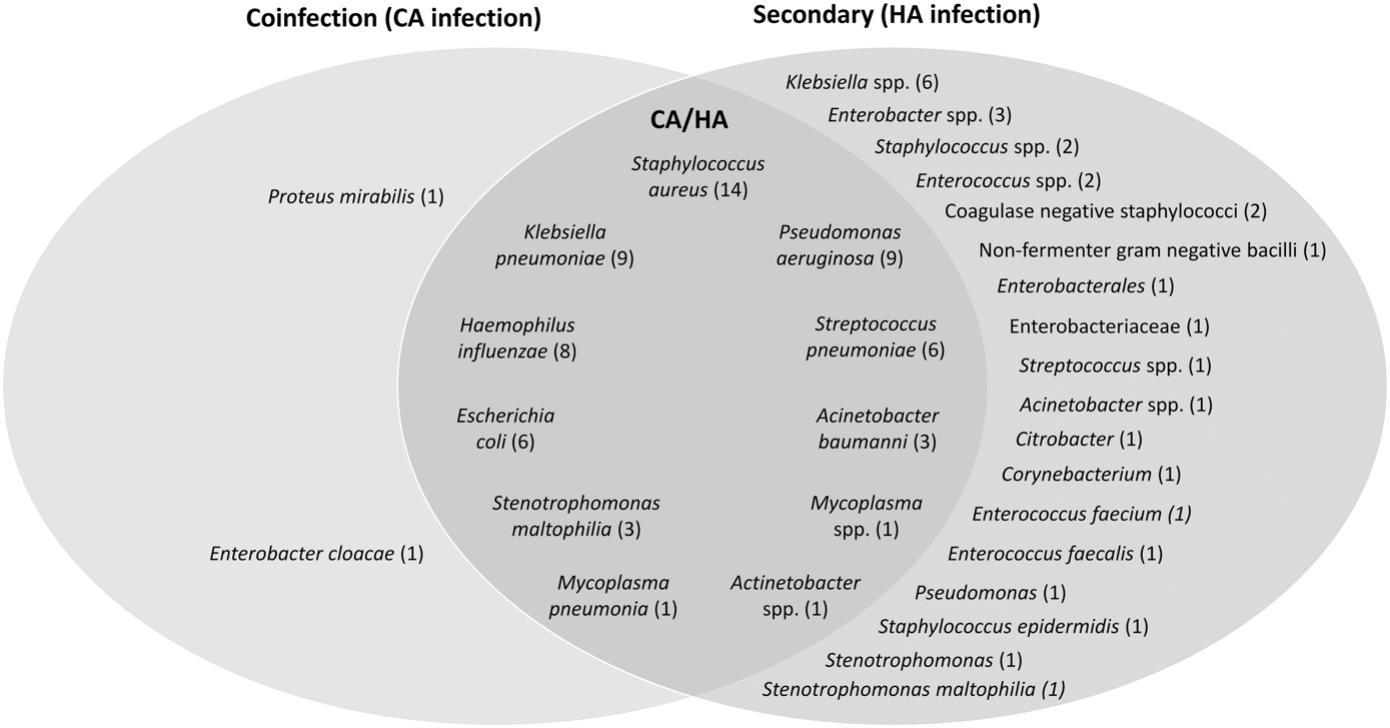
Common etiologies of bacterial coinfections and/or secondary bacterial infections in patients with COVID-19. The most frequently reported bacterial microorganisms from 22 studies (up to 5 of the most common bacterial microorganisms) were included for each type of infection from each study: 6 studies for CA infection,^17,18,21,27,36,63^ 12 studies for HA infection,^11,12,15,20,27,28,30,35–38,40^ and 6 studies for both CA and HA^22,24,26,32–34^ (studies could report >1 type of infection). The number of studies that reported each organism are shown in parenthesis. Note. CA, community-acquired; COVID-19, coronavirus disease 2019; HA, hospital-acquired.

Common microorganisms causing secondary bacterial infections and/or bacterial coinfections in patients with COVID-19 are shown in Figure 1. No clear pattern of preponderant pathogens was observed; however, the most frequently reported pathogens associated with both CA and HA infections were *Escherichia coli*, *Streptococcus pneumoniae*, *Staphylococcus aureus*, *Pseudomonas aeruginosa*, *Klebsiella pneumoniae*, *Haemophilus influenzae*, *Acinetobacter baumannii, Mycoplasma* spp, *M. pneumoniae*, *Stenotrophomonas maltophilia,* and *Acinetobacter* spp (Fig. 1).^22,24,26,27,32–34^ Notably, only 2 additional microorganisms were observed to only cause CA infection: *Enterobacter cloacae* (2 cases among 5 patients with bacterial respiratory infections)^18^ and *Proteus mirabilis* (18 (8%) of 221 cultures from 183 patients with COVID-19 and CA infections).^21^ Each was reported in 1 study. A wide range of other pathogens were reported to only cause HA infection across a range of studies (Fig. 1).^11,12,15,28,30,35–38^

In a retrospective study of 254 hospitalized patients with COVID-19, the proportion of pathogens detected increased with the duration of ICU stay, consisting mainly of gram-negative bacteria, particularly *K. pneumoniae* and *E. coli*.^36^ In contrast, *S. aureus* and *S. pneumoniae* were the pathogens most commonly detected within 48 hours of hospital admission.^36^ Similarly, a retrospective study of 3,028 hospitalized COVID-19 patients showed that the proportion of gram-negative bacteria causing HA infections increased with longer hospital stay, whereas staphylococci were more commonly isolated within the first 14 days of hospitalization.^21^ Beyond day 14 of hospitalization, Enterobacterales and *Pseudomonas* spp predominated.^21^ Overall, this finding reflects an increased acquisition of pathogens and wider range of organisms with length of hospitalization.

## Impact of secondary bacterial infections and bacterial coinfections on outcomes in patients with COVID-19

Mortality rates reported in patients with COVID-19 who had bacterial coinfections and/or secondary bacterial infections ranged between 6.5% and 66.7%; however, observation periods and population differed, which may account for the wide variation in rates (Tables 1–3).

In several studies, mortality rates were significantly higher in COVID-19 patients with bacterial coinfections or secondary bacterial infections compared with those without.^12,14,15,19,24,35,37–39^ Notably, in one study, high 14-day mortality rates (54.8%) and 30-day mortality rates (66.7%) were reported among 42 hospitalized patients with COVID-19 and *S. aureus* bacteremia.^25^ In a second study of 1,705 patients with COVID-19, mortality rates were significantly higher in patients with CA bacterial infections compared to those without (47.5% vs 18.0%; *P* < .001).^14^ However, in other studies, no difference in mortality rates between patients with and without bacterial coinfection or secondary bacterial infection was reported, with an overall mortality rate at 28 days of 31.5% in patients with COVID-19.^40,41^ These discrepancies may be due to low sample size in some studies, leading to inadequate power to detect a mortality difference.^40^ In other studies, most deaths occurred early during hospitalization; therefore, less time was available to collect microbiological samples.^41^ This finding suggests that the rate of bacterial infections in patients with COVID-19 might be underestimated.

Along with increased mortality, other noteworthy trends among COVID-19 patients with bacterial coinfection or secondary bacterial infection included prolonged length of hospital stay,^14,15,21,24,40^ more frequent ICU admission,^13,15,19,21,24,40^ and use of invasive mechanical ventilation.^21,24,28,38,40,42–45^ In a study of 100 COVID-19 patients, patients severely or critically ill at the time of admission were 4.4 times more likely to develop a bacterial infection,^24^ and those with bacterial infections were more likely to be admitted to the ICU compared with patients without bacterial infections (56% vs 18%; *P* < .001).^24^ Similarly, patients with CA bacterial pneumonia (CABP) were more likely to be admitted to the ICU compared with patients without coinfections (33% vs 16%; *P* < .01).^13^ Interestingly, in a single-center retrospective study of 989 patients, hospital length of stay was only significantly increased in patients with HA bacterial infections, and not in those with CA bacterial infections.^15^ Furthermore, other studies have also reported that patients with bacterial coinfections or secondary bacterial infections were older in age^13–15,17,21,33,36,38,46,47^ and were immunocompromised.^15,21,48^ These populations typically at greater risk of developing severe COVID-19 and frequently have chronic underlying conditions and comorbidities, such as diabetes, kidney disease, or cancer.^14,15,27^

Nasir et al^24^ reported that a larger proportion of patients with COVID-19 and bacterial infections received treatment with systemic steroids compared with patients without bacterial infections (92% vs 62% respectively; *P* = .001) and that treatment with steroids was a significant risk factor for bacterial infections.^24^ In a study of 226 hospitalized COVID-19 patients, treatment with steroids increased the risk of bacterial infections but steroid use did not affect the mortality rate (Table 3).^48^ In another study of 111 hospitalized COVID-19 patients, tocilizumab use was associated with patients with high risk of developing bacterial or fungal infections (Table 3).^47^ Although mortality in the group of patients who received tocilizumab was higher than those not receiving treatment (39.6% vs 17.4% respectively; *P* = .016),^47^ this may be due to the fact that patients in the tocilizumab group were sicker and tocilizumab use predisposes to secondary bacterial infections.

## Antibiotic treatment approaches in patients with COVID-19 and secondary bacterial infections or bacterial coinfections

**Fig. 2.**
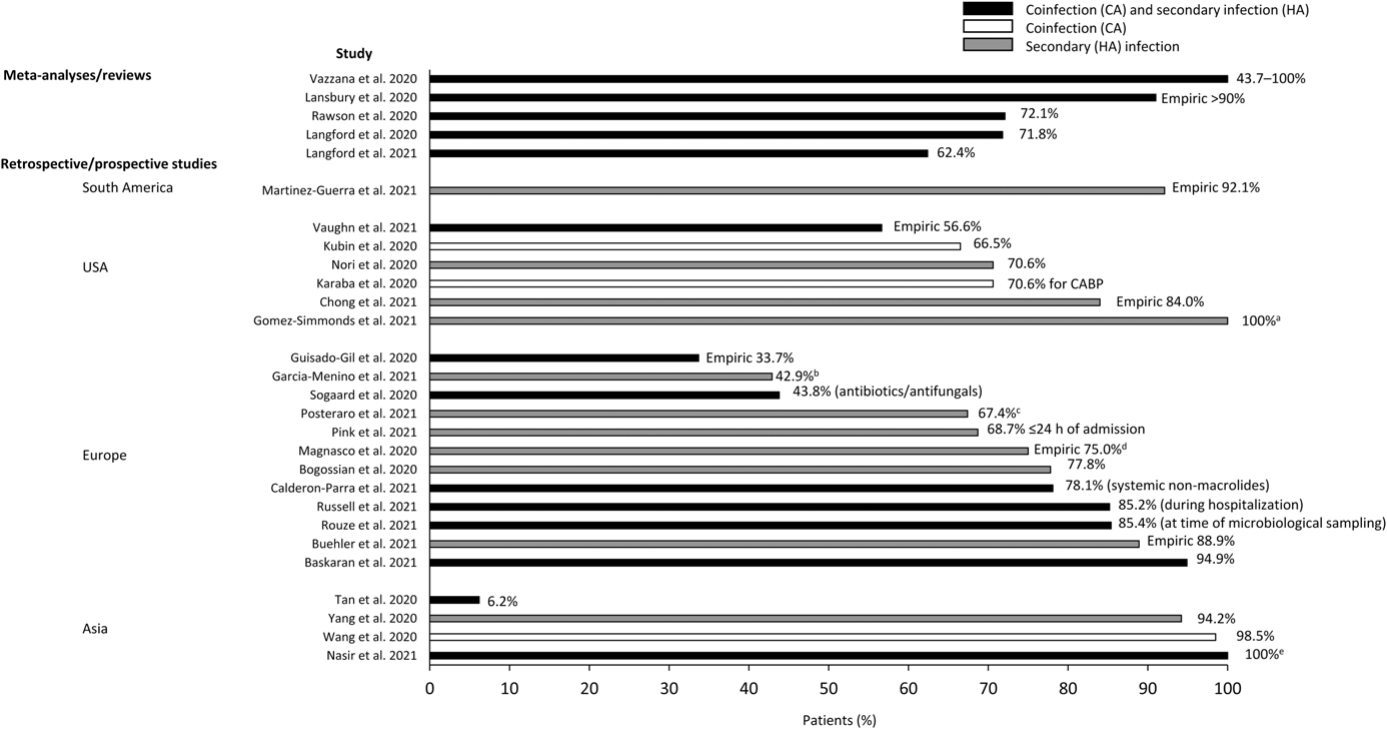
Proportion of patients with COVID-19 receiving antibiotics: (a) in patients with COVID-19 and carbapenemase-producing Enterobacterales; (b) in patients with carbapenem-resistant *Klebsiella pneumoniae*; (c) in patients with COVID-19 and bloodstream infection; (d) in patients with carbapenem-resistant *Pseudomonas aeruginosa* for suspected bacterial superinfection; and (e) in patients with COVID-19 and bacterial infection. Note. CA, community-acquired; COVID-19, coronavirus disease 2019; HA, hospital-acquired.

**Fig. 3.**
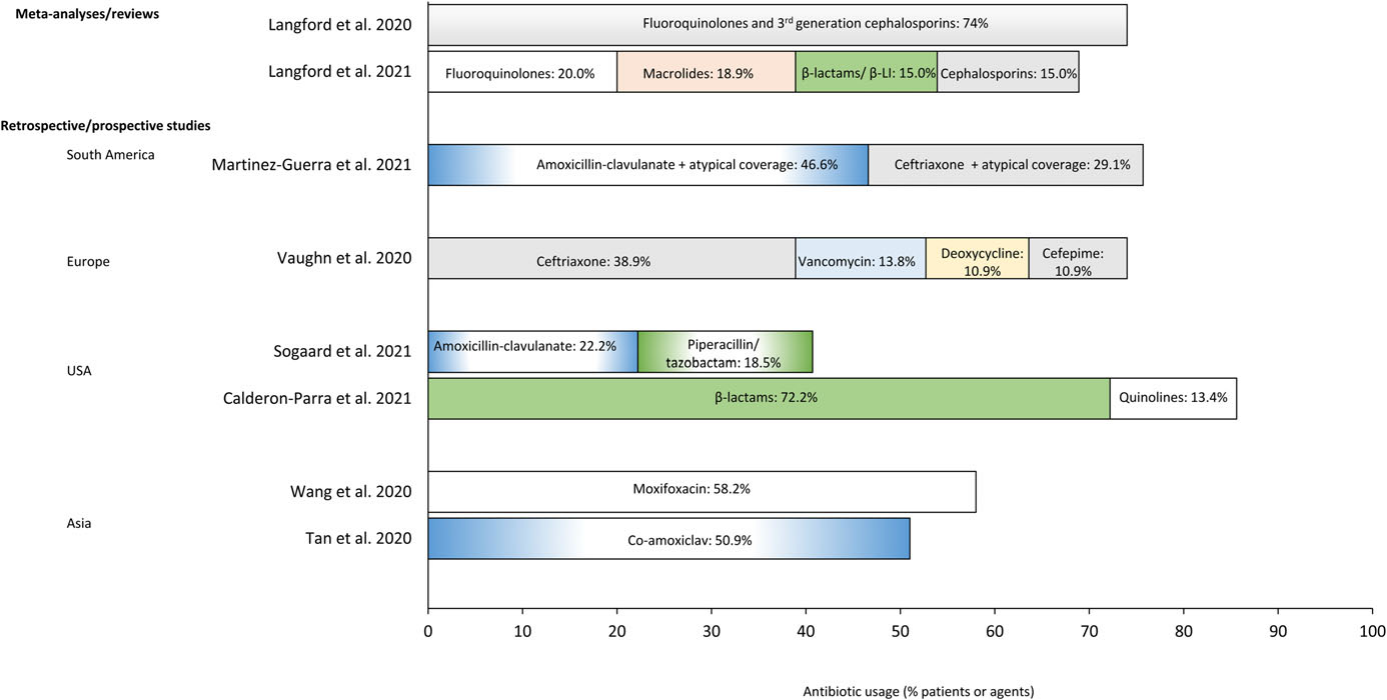
Most frequently used antibiotics in patients with COVID-19.^a^ (a) Studies expressed data as percentage of patients receiving antibiotic treatments, except the study by Langford et al,^52^ in which data were presented as percentage of prescriptions of an antibiotic class per total number of antibiotic prescriptions. Data are provided only for antibiotic classes that were used in >10% of patients or > 10% of prescriptions. Note. β-LI, β-lactamase inhibitors; COVID-19, coronavirus disease 2019.

Considerable heterogeneity in reported treatment rates and antibiotic treatment approaches was reported across the studies included in this review, perhaps in part due to the variability in study locations and differing local and national guidelines to antibiotic treatment (Fig. 2 and Fig. 3). National US guidelines (from the National Institutes of Health), updated in April 2021, recommend empiric antibiotics if secondary bacterial pneumonia or sepsis is suspected in patients with COVID-19 but to re-evaluate patients daily and de-escalate or stop antibiotic treatment if there is no evidence of bacterial infection.^49^

Despite the low rates of secondary bacterial infections observed, most studies reported the use of empiric antibiotic treatment, with 33.7% to > 90% of COVID-19 patients treated (Tables 2 and 3).^12,30,34,40,50,51^ However, data are limited and information was not available on the duration of treatment. Although many patients did not have a confirmed bacterial infection at the start of treatment, data were not available on patients who stopped or altered treatment once microbial testing to confirm bacterial infection was performed. The variation in the range of patients receiving antibiotics could be explained by the differences in geographic location, the diversity of the populations treated, the time when studies were done, and so on.

These findings suggest that antibiotic utilization was high in patients who did not have bacterial infection. In a study of 48 COVID-19 patients, no significant difference was reported in the use of empiric antimicrobial therapy in critically ill patients either with bacterial superinfection (88%) or without bacterial superinfection (94.7%).^30^ Notably, all studies that included data on both infection rates and antibiotic use reported mismatch between use of antibiotics versus confirmed secondary or coinfection, regardless of whether infection was CA or HA (see Tables 1–3 and Fig. 2).^12,14,21^ In a meta-analysis of patients with COVID-19, the prevalence of antibiotic prescribing was 62.4%, whereas the estimated rate of bacterial coinfection was 8.6%.^52^ In a systematic review reporting bacterial and fungal coinfections in 806 patients with COVID-19, 72.1% received antimicrobial therapy despite only 8% of patients having bacterial or fungal coinfections during their hospitalization.^31^

A recent meta-analysis of antibiotic prescribing in 30,623 patients with COVID-19 reported considerable heterogeneity across regions with a prevalence of 63.1% (95% confidence interval [CI], 41.7%–80.4%) in Europe, 64.8% (95% CI, 54.0%–74.2%) in the United States, 76.2% (95% CI, 66.8%–82.3%) in China, 86.0% (95% CI, 77.4%–91.7%) in the Middle East, and 87.5% (95% CI, 47.8%–98.2%) in East and Southeast Asia (excluding China).^52^ Only 5 (3.2%) of 154 studies included in this meta-analysis provided data on duration of antibiotic treatment.^52^ Antibiotic stewardship strategies were reported in 3 studies (1.9%), including recommendations to avoid antibiotics in patients without suspected coinfection (n = 2) or to de-escalate antibiotics when additional data became available (n = 1).^52^ In a retrospective study of 13,932 hospitalized patients with COVID-19 who were prescribed antibiotics in 150 hospitals in Spain from March 1 to June 23, 2020, antibiotics were prescribed for respiratory bacterial coinfections and/or secondary infections in 10.9% of patients with COVID-19 and 43.8% of total antibiotic prescriptions were considered inappropriate.^53^ Interestingly, younger age and fewer comorbidities were independently associated with inappropriate antibiotic prescribing.^53^ Notably, a lower percentage of inappropriate antibiotic prescribing was observed in patients hospitalized after March 2020 in this study, which suggests increased awareness of the problem among healthcare professionals and a better understanding of the disease.

The types of antibiotics prescribed differed across the studies we reviewed, although most were broad-spectrum agents, including fluoroquinolones, β-lactam and β-lactamase inhibitors, cephalosporins, macrolides, and penicillin-like agents (Fig. 3). This pattern of antibiotic prescribing likely reflects the empirical use of these agents, which tends to provide coverage of multiple organisms while awaiting culture results or confirmation of coinfection or secondary infection.

The potential overuse or misuse of antibiotics in the context of the COVID-19 pandemic could contribute to increased AMR.^54^ AMR has been widely reported, including infections with multidrug-resistant (MDR) organisms,^15,16,19,28,30,35,37,42^ and methicillin-resistant *Staphylococcus aureus*.^25,32,34–36,40,48^ In a study of 989 COVID-19 patients, MDR gram-negative bacteria were isolated in 7 of 43 patients with HA infections: 3 had MDR *P. aeruginosa* infection, 2 had extended-spectrum β-lactamase *E. coli*, and 2 extended-spectrum β-lactamase *K. pneumoniae*.^15^ Søgaard et al^16^ only reported 1 MDR pathogen (*Acinetobacter baumannii*, Oxa-23) isolated in a case transferred from a hospital abroad. Buehler et al^30^ reported that MDR bacteria (*Pseudomonas aeruginosa*, *Enterobacter cloacae*, and *Burkholderia cepacia*) were detected in 22.2% of all hospitalized COVID-19 patients.

Increased AMR leads to high exposure to antibiotic treatments, which can have detrimental consequences and can facilitate subsequent infections during ICU stay, particularly by gram-positive pathogens such as enterococci.^55^ In a US cohort study of hospitalized patients with sepsis, inadequate broad-spectrum empiric antibiotic treatment was associated with ICU hospitalization and increased mortality.^56^ Interestingly, inadequate antibiotic therapy was 4 times more likely in patients with resistant pathogens (eg, methicillin-resistant *Staphylococcus aureus*) than with nonresistant pathogens (*P* < .001), older patients, and patients with comorbidities.^56^ Thus, improved treatment strategies (antimicrobial stewardship) and treatment options with newer antibiotics that have lower resistance rates are needed.

## Antibiotic stewardship perspectives

The incidence of secondary bacterial infections and bacterial coinfections in patients with COVID-19 is relatively low, with lower rates of CA bacterial infections than HA infections. The incidence and variety of infecting pathogens increased with the length of hospitalization. Overall, the rates of secondary bacterial infections and bacterial coinfections in patients with COVID-19 were lower than rates of secondary bacterial infection and/or coinfection associated with other viral respiratory diseases such as influenza.^57^ The relatively low incidence of bacterial coinfections and/or secondary infections reported during the COVID-19 pandemic could be a consequence of the implementation of national lockdowns and social distancing measures adopted by many countries during the pandemic, as was suggested in an international study demonstrating that COVID-19 lockdowns significantly reduced transmission of *S. pneumoniae*, *H. influenzae*, and *N.meningitidis*, leading to significant reductions in life-threatening invasive diseases worldwide.^58^

Despite the relatively low rates of bacterial secondary infections and/or bacterial coinfections observed during the COVID-19 pandemic, high percentages of patients have been receiving antibiotic treatment. Empiric treatment was common, perhaps because COVID-19 patients are often hospitalized during the hyperinflammatory phase of the disease, making differentiation between viral and secondary bacterial infections challenging.^59^ From a clinical perspective, the mismatch between antibiotic utilization and reported rates of bacterial infection is of particular concern because it may exacerbate the development of AMR and associated complications.

Increased empiric antibiotic prescribing may have been due to the diversion of stewardship efforts to pandemic responsibilities and away from core activities.^60^ Investigation of the optimal antimicrobial stewardship program interventions into pandemic response efforts to limit antibiotic overuse is warranted. However, despite national guidelines aiming to rationalize antibiotic use and maintain safe medication use in the ICU,^49,61,62^ the emergency caused by the COVID-19 pandemic probably made it difficult to apply these guidelines, with overwhelmed wards and ICUs and busy healthcare professionals. Moreover, the diagnosis of bacterial infections remains a challenge, and it is difficult to distinguish between severe viral pneumonia and bacterial infection. Microbiological investigations, which are not routinely performed in patients with COVID-19, take several days to result and do not differentiate bacterial colonization from infection.^14^

Thus, the pandemic may have a lasting impact on AMR, and the long-term impact on antibiotic overuse during COVID-19 pandemic remains to be seen. The data reported here show multidrug-resistance pathogens and indicate that current empiric treatment strategies may not be effective. The development of newer antibiotics is urgently needed, particularly considering the increase in multidrug resistance for which there are no treatment options.

Although a strength of this review is the use of a comprehensive search strategy, several limitations must be considered. First, most of the included studies were small, retrospective, observational studies, with a large degree of heterogeneity between them in terms of patient populations, geographic locations, and treatment protocols. Many of the included studies lacked consistent bacteriological diagnostic and specific testing upon patient admission to hospital, which likely affected stratification of CA versus HA infections. Some studies did not give precise details regarding the timing of diagnosis, making the differentiation between CA and HA challenging. Finally, most studies included in this review were from Asia, Europe, and North America (United States), and regional differences in the patient populations, access to care, clinical practices among hospitals, and patient follow-up must be considered.

To conclude, recent data indicate that secondary bacterial infections and bacterial coinfections in patients with COVID-19 are associated with worse patient outcomes. Importantly, antibiotic utilization was consistently higher than bacterial infection rates, highlighting the need to improve appropriate treatment approaches to mitigate the complications of the misuse of antibiotics. Furthermore, due to the incidence of multidrug-resistant bacterial pathogens, new treatment and antibiotics that could overcome the problem of resistance are urgently needed. Implementing and following stewardship programs will be of crucial importance to prevent the development of resistance and to improve patient outcomes.

## Supplementary material

For supplementary material accompanying this paper visit https://doi.org/10.1017/ash.2022.253. click here to view supplementary material
