# Supplementary material for: Coronavirus disease 2019 (COVID-19): Secondary bacterial infections and the impact on antimicrobial resistance during the COVID-19 pandemic
Source: Antimicrob Steward Healthc Epidemiol. 2022 Jul 11;2(1):e114. doi: 10.1017/ash.2022.253 (PMC9726554; doi:10.1017/ash.2022.253)
Supplement: Supplementary file 1 [file S2732494X22002534sup001.docx]

**Supplementary Table 1.** Literature search strategy

| **Search area** | **Search String - PubMed** | **Search String - Embase** |
| --- | --- | --- |
| Bacterial co-infection/secondary infection including pneumonia in COVID-19 patients | (Coronavirus[Title/Abstract] OR COVID-19[Title/Abstract]) AND (secondary bacterial infection OR secondary coinfection OR secondary co-infection]) AND bacterial pneumonia | (ti(“coronavirus” or “COVID-19”) AND ab(“secondary bacterial infection”) AND ab(“bacterial pneumonia”) NOT medl(yes)) |
|  | (Coronavirus[Title/Abstract] OR COVID[Title/Abstract]) AND (secondary bacterial infection OR secondary coinfection OR secondary co-infection) AND (rate OR incidence) | (ti("coronavirus" OR "COVID-19") AND ab("antimicrobial resistance" OR "antibiotic resistance")) |
| AMR in COVID-19 patients | (Coronavirus[Title/Abstract] OR COVID-19[Title/Abstract]) AND ((antimicrobial resistance[Title/Abstract])OR (antibiotic resistance[Title/Abstract])) | (((ti(“coronavirus” or “COVID-19”) AND ab("secondary bacterial infection") AND ab("rate" OR "incidence")) NOT medl(yes))) |
| Bacterial pneumonia in COVID-19 patients | (Coronavirus[Title/Abstract] OR COVID[Title/Abstract])AND (secondary bacterial pneumonia[Title/Abstract]) | (ti(“coronavirus” or “COVID-19”) AND ab(“secondary bacterial pneumonia”) NOT medl(yes)) |

Inclusion criteria for the search were: English language; studies of adult humans; reviews, clinical trials, meta-analyses and guidelines; secondary bacterial infections, particularly secondary bacterial pneumonia; community-acquired bacteria pneumonia infections; incidence of secondary bacterial infection, mortality rate, hospital utilization, antibiotic treatment, failure rates and outcomes.

Exclusion criteria for the search were: Any language other than English; pediatric studies, animal studies, editorials, responses, case reports, comments, or congress abstracts; non-bacterial infections (viruses, fungi); ventilator associated (VAP) or hospital associated (HAP) pneumonia infections; no relevant data.
